# Looking in the mirror: situs inversus totalis

**DOI:** 10.11604/pamj.2015.20.87.6139

**Published:** 2015-01-29

**Authors:** Anastasia Paschala, Theocharis Koufakis

**Affiliations:** 1 Department of Internal Medicine,St. Josef Hospital, Gelsenkirchen Horst, Germany; 2 Department of Internal Medicine, General Hospital of Larissa, Larissa, Greece

**Keywords:** Situs inversus, dextrocardia, congenital syndrome

## Image in medicine

A 50 year-old male presented to the Emergency Department with complaints of abdominal pain. His chest and abdomen X-rays demonstrated some unexpected findings: dextrocardia (A) and right-sided stomach bubble (B). Further evaluation of the patient revealed a urinary tract infection as the cause of his symptoms. Situs inversus totalis is defined as the complete inversion of the thoracic and abdominal organs, which constitute a mirror image of the normal anatomy. It was first described by Matthew Baillie in the 16^th^ century and is a rare, congenital condition, with an overall frequency estimated to be approximately at 1/10000 live births. The syndrome has a genetic background, following an autosomal recessive pattern of inheritance. Subjects with the condition are usually asymptomatic, as the structure and function of vital organs are generally unaffected. However, in 25% of the cases, the syndrome coexists with primary ciliary dyskinesia (Kartagener syndrome), then characterized by chronic sinusitis, bronchiectasis and susceptibility to infections. A random X-ray test for an unrelated condition is often the occasion that leads to the diagnosis. The clinical significance of situs inversus totalis relates to the fact that, due to the reversal of the inner organs, symptoms and signs elicited during patient's physical examination are present on the atypical side of the body.

**Figure 1:**
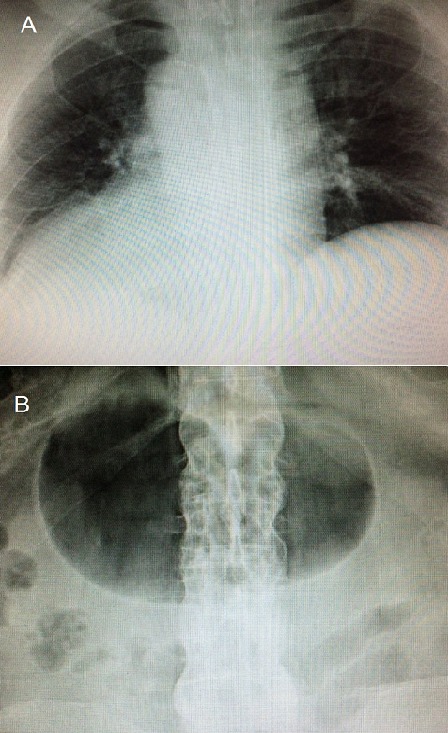
(A) chest X-ray showing right-sided aortic arch and cardiac apex pointing to the right; (B) abdomen X-ray demonstrating a dilated right-sided stomach bubble

